# Evolution and vulnerability analysis of global photovoltaic industry chain trade pattern

**DOI:** 10.1038/s41598-025-90234-6

**Published:** 2025-02-21

**Authors:** Chao Ding, Xiaobei Ren

**Affiliations:** 1 School of Management, Hebei GEO University, No. 136 Huai’an East Road, Yuhua District, Shijiazhuang City, 050031 Hebei China; 2 Strategy and Management Base of Mineral Resources in Hebei Province, Hebei GEO University, Shijiazhuang, 050031 China

**Keywords:** Photovoltaic industry chain, Vulnerability, Kernel density estimation, PageRank centrality, Environmental economics, Sustainability

## Abstract

As global energy demand increases, photovoltaic power generation has become the solution to the energy conundrum. Based on global photovoltaic product trade data from 2000 to 2023, this paper examines the development of photovoltaic industry chain trade pattern and impact of PageRank centrality top 10% node failures on network vulnerability. It accomplishes this by employing hypothetical assault strategies and intricate networks. Research has shown that: ① The trade pattern of PV industry chain has undergone profound changes. The trend of multipolarization is becoming more and more obvious. China’s position in each link of the industry chain is increasingly prominent. ② The vulnerability of the PV industry chain network shows a weakening trend. The vulnerability of upstream and midstream networks is more stable. The destruction resistance of the downstream network has been improved. Fluctuation of the node impact on the network structure has weakened. ③ There is a distinct gradient of susceptibility in PV industrial chain. The vulnerability of the downstream is relatively low, followed by the midstream. The vulnerability of the upstream is relatively high.

## Introduction

The Paris Agreement was signed by 196 countries in 2015. It encourages countries to develop renewable and clean energy sources, making energy security a top priority. Energy security has been recognised as one of the biggest threats globally ^1^. The concept of energy security was first introduced in 1975 and has only begun to receive attention in the early twenty-first century^2^. Energy security is defined as ensuring the stability and reliability of energy supply at affordable prices while avoiding irreversible damage to the environment and ecology^3^. Energy security consists of two major aspects: security of energy supply and security of energy use. Security of energy supply emphasises ensuring a stable, reliable and sustainable supply of energy to meet the needs of the country’s economic and social development. Security of energy use focuses on the efficient, clean and environmentally friendly use of energy to reduce pollution and damage to the environment. However, the COVID-19, Russia-Ukraine conflict, the restart of coal power in Europe and a series of other changes in the global energy stability and security has brought a lot of new variables^4,5^. The topic of energy security has grown in importance and drawn the attention of both governments and scholars.

In the face of the depletion of traditional energy sources and the aggravation of environmental pollution, the search for new energy solutions has become an urgent need to guarantee energy security. Photovoltaic (PV) power generation, as a clean and renewable form of energy, provides a new way of thinking to address the energy security problem. Solar energy is one of the fastest growing renewable energy sources since 2013^6^. The photovoltaic industry directly utilizes solar energy which is a virtually endless resource. It is not affected by geopolitical conflicts or resource depletion and enhances the security of energy supply. The photovoltaic industry is green, efficient and sustainable, which can guarantee the security of energy use. To start with, the wide application of photovoltaic products effectively reduces the dependence on fossil fuels and lowers greenhouse gas emissions. In its energy conversion process, it does not produce harmful substances and is friendly to the environment. Furthermore, the PV industry usually adopts modular design, which can be flexibly configured according to different energy demands and site conditions, thus optimizing the efficiency of energy use. At the end, it can also promote the transformation of energy structure and the sustainable development of society.

In recent years, the trade of PV industry has shown a booming trend. Global trade volume of PV products has continued to grow, increasing from US$33 billion in 2000 to US$190.7 billion in 2023. More and more countries and regions participate in the trade of the PV industry. In 2023, nearly 240 countries and regions participate in one after another. Trade links of the PV industry will become closer and closer. The main trade object covers many countries such as China, Germany, Japan, the United States and so on. Especially China, as the world’s largest producer and exporter of PV products, it plays a pivotal role in the global PV industry chain. However, there are many challenges hidden behind the prosperity of PV industry trade. Geopolitical conflicts, trade barriers, technical blockades and other issues are becoming increasingly prominent. Stability and resilience of the global photovoltaic industry chain is facing a severe test. United States and other countries have taken unilateralist measures and imposed high tariffs and technical restrictions on PV products. This further aggravates the uncertainty of trade in the PV industry.

Therefore, this study explores the evolution of the trade pattern of the global PV industry based on complex network theory. It identifies key nodes and analyzes the impact of the exit of key nodes on the vulnerability of the PV industry chain. This helps to identify and respond to potential risks in advance. The government can use this as a crucial guide when creating and modifying pertinent policies. This encourages the PV industrial chain to grow in a more constructive and wholesome manner.

## Literature review

Academic study on the trade of the photovoltaic industry currently primarily examines the viability of pertinent policies, economic implications and motivating factors, and the choice of implementation strategy. The efficacy evaluation of the photovoltaic sector subsidy policy was primarily conducted in terms of the viability of pertinent policies. According to Shi et al. (2023), the photovoltaic industry’s subsidy program remains effective in an open economy^7^. When Zhu et al. (2011) evaluated the policies of China, Germany, and Japan in the photovoltaic industry, they discovered that the projected effects needed to take into account the type of policy tools and the flexibility of the policy environment^8^. In terms of driving factors and economic consequences, a large number of scholars have found that trade in the photovoltaic industry can easily trigger geopolitical conflicts between countries. Goldthau et al. (2020) found that countries can have a profound impact on geopolitical relations by leveraging their advantages in the energy sector^9^. Yang et al. (2022) pointed out that renewable energy has become a key factor in triggering geopolitical conflicts between countries. The important factors affecting photovoltaic trade are trade frictions and conflicts^10^. McCarthy et al. (2016) found that anti-dumping investigations initiated by the European Union provided a protective effect for their enterprises in the short term^11^. While, this protection was far from sufficient to compensate for their actual losses. Instead, it encouraged the Chinese photovoltaic industry to reorganize its domestic and international business, accelerate the industry’s self adjustment and optimization. Zhu et al. (2018) found that the trade protection policies of European and American countries have not been able to successfully suppress the development of China’s photovoltaic industry^12^. In contrast, it reshapes the pattern of China’s photovoltaic industry. In terms of path selection,scholars generally believe that the "the Belt and Road" is an important driving force for the development of photovoltaic industry. Yang (2016) believes that the market should be expanded with the help of the “the Belt and Road”^13^. Trade zone should be established to optimize trade. International logistics network should be used to improve transport efficiency. We also need to promote the common development of photovoltaic industry with countries along the line and achieve mutual benefit and win–win results. Yu et al. (2020)pointed out that in order to achieve the development of photovoltaic industry, we must give full play to the comparative advantages of endowments and carry out echelon transfer to countries at different levels of the "the Belt and Road"^14^.

The majority of the current research on the trade of the photovoltaic industry chain is concerned with the traits of the trade pattern’s evolution. For instance, Ding et al. (2024) contrasts the trade patterns of the upstream and midstream links of the solar industry chain^15^. The study discovered that: the middle reaches’ trade scale is still growing, creating a bipolar trade pattern centered on China and the United States. The trade scale of the upstream links gradually shrank. Trade network relationships were sparse. Trend of trade concentration was evident. China is occupying an absolute core position.The trend of “core periphery” has always been evident. Position of emerging countries such as Southeast Asia in the network is beginning to emerge.

The concept of vulnerability was proposed by Timmerman in 1981, referring to the degree of damage that a system may suffer in the face of unfavorable factors, as well as its ability to recover from damage^16^. Vulnerability research originally originated in the field of natural disasters^17^. It used to analyze the impact of natural disasters on human society. It also concerns how to reduce losses by improving system vulnerability. As research deepens, vulnerability research is not limited to disaster emergency management^18^, but gradually expands to broader fields such as ecology^19^, public health^20^, climate change^21^, supply chain management^22^. With the in-depth study of complex systems, scholars are aware that vulnerability is closely related to its overall structure, connectivity, and dynamic characteristics. Consequently, complex network theory and methods are gradually introduced into the study of network vulnerability.

The current research on network vulnerability mainly focuses on transportation and shipping networks. For example, Li Bo et al. (2023) studied the vulnerability of Shaanxi Province’s highway network. The results showed that the network is vulnerable to node or edge attack strategies^23^. Mei Q et al. (2024)evaluated the vulnerability of the European liquefied natural gas maritime supply chain network. He found that the vulnerability of the network increased in 2020 compared to before^24^. Meanwhile, a small number of scholars have focused their research on the vulnerability of mineral resource trade networks such as lithium^25^, rare earths^26^, and tantalum^27^.

The aforementioned study shows that while there is comparatively little research on photovoltaic industry chain trade, the majority of research that is now available focuses on the development of photovoltaic industry trade. Two primary areas of network vulnerability research are transportation networks and mineral resources trading networks. Research on renewable energy’s vulnerability is lacking, particularly with regard to the solar industry chain trade network. Simultaneously, current research mostly concentrates on the vulnerability of networks lasting a single year, disregarding the analysis of networks with multiple years of vulnerabilities. As a result, this study uses the solar industry chain as its starting point, identifies important network nodes and models how the network’s vulnerability evolves in the event of a trade disruption. This offers solid assurances for the security of the global energy supply and opens up new avenues for in-depth study on photovoltaic industry.

## Research methods and data sources

### Research methods

#### PageRank centrality

Google created the PageRank centrality algorithm in the beginning to rank web pages and determine where they would appear in search results. The fundamental idea is to mimic the actions of random walkers inside the network and use recursive methods to determine each node’s PageRank value. This algorithm has been quite successful in the search engine industry and has progressively spread to other industries, including social networks and trade networks. This metric not only considers the direct linking of nodes, but also comprehensively evaluates the indirect influence of nodes through an iterative algorithm. It more accurately identifies the critical nodes and critical paths in the network.

Two popular techniques for identifying critical nodes are PageRank centrality and social network analysis. Social network analysis (It includes degree centrality, proximity centrality, betweenness centrality. Degree centrality measures the number of connections between nodes. Proximity centrality reflects the average distance between nodes and other nodes. Betweenness centrality is the number of shortest paths through a node.) is useful for showing a single node’s position within a network. On the other hands, it has drawbacks in that it leaves out the important "closer the red" effect in trade networks—the mutual influence and interaction between nodes. This article presents the PageRank centrality index. PageRank centrality indicates the importance or influence of a node in the network. It can identify the important nodes of the network precisely. The formula is as follows:

1 $$P{\text{age}}Rankit = \frac{1 - \alpha }{N} + \alpha \sum\limits_{j = 1}^{N} {Aji\frac{PageRankjt}{{Outjt}}}$$

Among them, PageRank_it_ represents the PageRank centrality of node i in year t, N represents the total number of nodes. A_ji_ represents the directed node transition matrix, Out_jt_ represents the outgoing degree of node j in year t, and α is the initial weight of each node, calculated using the power iteration algorithm.

#### Kernel density estimation

Kernel density estimation, as a nonparametric testing method, effectively estimates the probability density function through the distributional characteristics of the data. It shows a wide range of application value in analyzing the dynamic evolution of unbalanced distribution. This paper thoroughly examines the spatial distribution density, evolution trend, and extension status of important nodes using the kernel density estimation method. This is the precise formula:

2 $$P(x) = \frac{1}{Nh}\sum\limits_{i = 1}^{N} {K(\frac{Xi - x}{h}} )$$

Among them, P (x) represents the kernel density distribution function, N is the total number of nodes, h is the bandwidth, K(·) is the kernel function, X_i_-x represents the difference between the i-th node and the average value.

#### Attack strategy

Currently, there are two primary methods of attacking: targeted assaults and random attacks. Random attacks refer to chooses nodes in a network at random for elimination or destruction. Regardless matter how important a node or edge is to the network, there is an equal chance that it will be attacked. It primarily shows up as the network’s reaction to random events. A deliberate assault is when a node or edge in a network is eliminated or destroyed because of its qualities or significance. It applies mainly to the impact on the network of perturbations caused by trade conflicts, economic sanctions. Intentional attacks were selected as the attack tactic since the study’s focus is on how network vulnerability evolves following trade disruptions at important nodes. The attack object was the solar industry chain trade network built in 2023, and the study focused on the changes in network characteristic values following the failure of the top 10% of node nodes. The effect of the departure of important node countries on the vulnerability of the solar industry chain network was revealed by the change rate of network characteristic values following node failure. This helps us to better understand the operation mechanism and risk points of the PV industry chain network, and provides a scientific basis for formulating effective risk prevention and control measures.

#### Vulnerability assessment

Drawing on previous literature, this article selects the average clustering coefficient and network efficiency to reflect the vulnerability characteristics of the global photovoltaic industry chain:

① The average clustering coefficient (C) of the overall network is defined as the average of the clustering coefficients of all nodes in the network. The clustering coefficient of a single node is used to describe the degree of interconnection between its neighboring nodes, and the calculation formula is as follows:

3 $$C = \frac{1}{{\text{N}}}\sum\limits_{i = 1}^{N} {\frac{2mi}{{ki(ki - 1)}}}$$

Among them,C represents the average clustering coefficient, N is the total number of nodes, k_i_ is the number of adjacent nodes of node i, and m_i_ is the number of edges between adjacent nodes of node i.

② The efficiency of network operation (E) represents the efficiency of the network and is an indicator of the efficiency of information transmission between nodes in the network. It is usually defined as the average of the reciprocal of the shortest path length between all node pairs in the network. The calculation formula is as follows:

4 $$E = \frac{1}{N(N - 1)}\sum\limits_{i = 1}^{N} {\sum\limits_{j = 1(i \ne j)}^{N} \frac{1}{dij} }$$

Among them, d_ij_ is the shortest distance between node i and node j.

### Data sources

As for the products found in each link in the chain of the photovoltaic sector, current research has not yet resulted in a consensus. Six goods from 2000 to 2023 were chosen as representations of upstream, midstream, and downstream based on the research of Ding et al. (2024)^15^, Tian Yue (2022)^28^ and the data that was available (see Table 1 for details). Python is used to combine the data in this article, which comes from the United Nations Commodity Trade Database (UN Comtrade). This article did not filter the data in order to guarantee its integrity. Additionally, there are differences in the volume of commerce between importing and exporting nations as a result of the various statistical techniques employed by various nations. Nonetheless, prior research has demonstrated that data on import trade volume is more reliable^29^. Hence the data presented in this article comes from importing nations. The data scope of “China” referenced in this article refers to the Chinese Mainland because the data of Hong Kong, Macao, and Taiwan are published individually.

**Table 1:** Products and codes of each link in the photovoltaic industry chain.

Product Name	HS code	Industrial chain
polysilicon	280461	Upstream
Photovoltaic modules	850140	Midstream
Photovoltaic glass	700719	
Photovoltaic generator set	850239	Downstream
Photovoltaic inverter	850440	
Intelligent photovoltaic combiner box	853710	

## Result analysis

### Analysis of the evolution of global photovoltaic industry chain trade pattern

#### Evolution of trade scale in photovoltaic industry

**Fig. 1:**
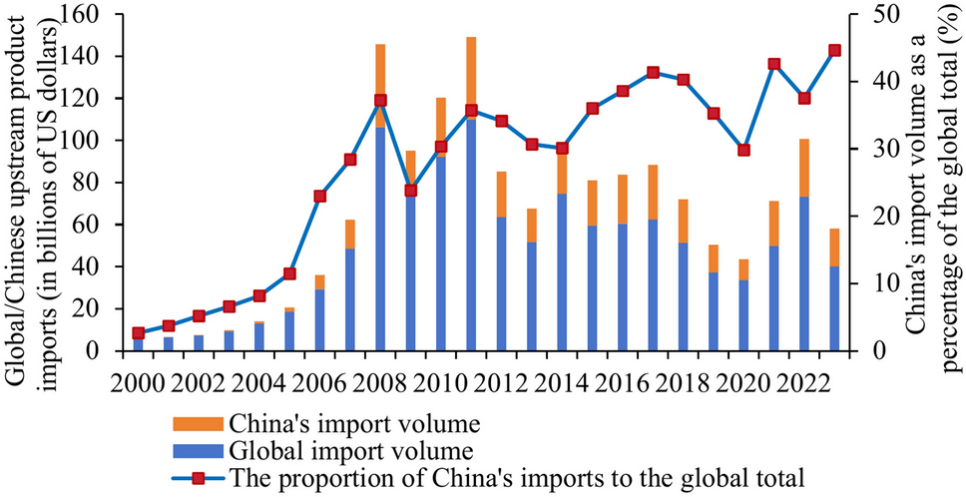
Global/China photovoltaic industry upstream product import trade volume.

**Fig. 2:**
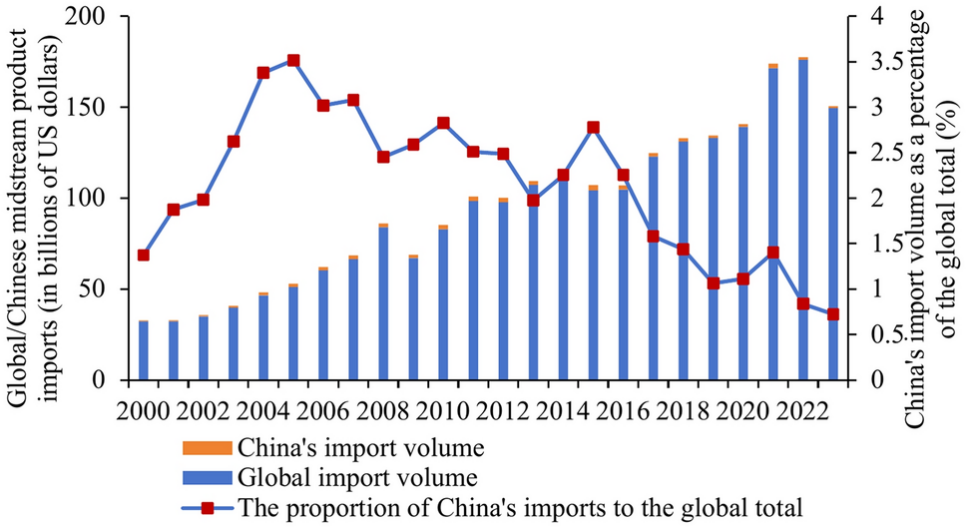
Global/China photovoltaic industry midstream product import trade volume.

**Fig. 3:**
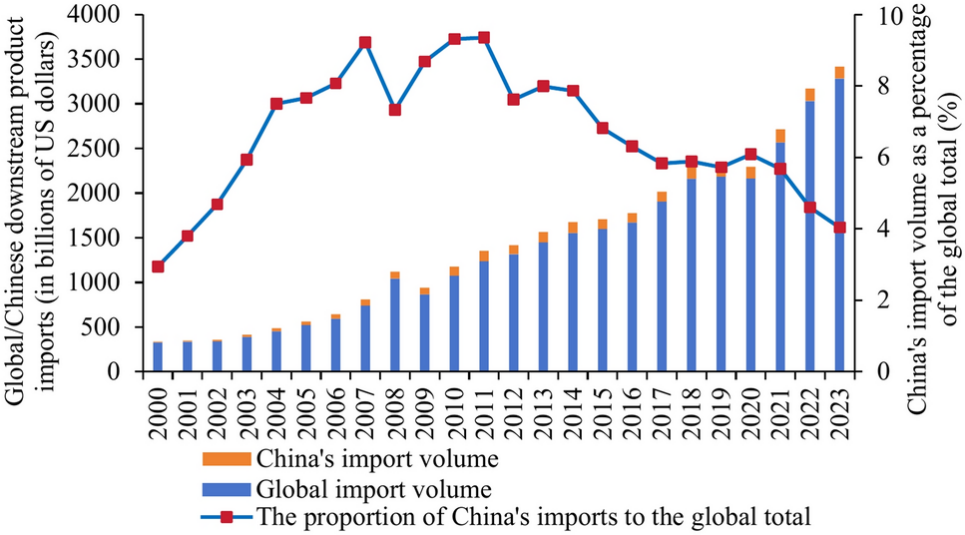
Global/China photovoltaic industry downstream product import trade volume.

Firstly, from 2000 to 2023, the import trade volume of upstream and midstream products in the global photovoltaic industry experienced a trend of first increasing and then decreasing (see Figs. 1 and 2). The import trade volume of downstream products showed a sustained growth trend (see Fig. 3). Behind this phenomenon, it is closely related to the development process and technological changes of the photovoltaic industry. In the early stages of the photovoltaic industry before 2012, due to immature technology, the manufacturing costs of upstream and midstream product were relatively high. Therefore, most nations with somewhat outdated photovoltaic technology have opted to import these items in order to meet domestic demand. However, since solar technology advances quickly, an increasing number of nations are starting to be able to create upstream and midstream goods on their own. This has reduced the need for imports of these goods. For downstream products, with the increasing emphasis on renewable energy, the global demand for photovoltaic products continues to rise. Especially in developing countries, there is a huge demand for infrastructure construction. The investment and construction efforts in photovoltaic power plants are also continuously increasing. Imports of downstream products are stimulated. This directly leads to the ongoing growth of the volume of downstream product import trade.

Secondly, the import trade value of each link of China’s PV industry chain as a whole shows a fluctuating upward trend. Especially the upstream products, China’s import trade volume accounted for the global proportion of the growth is particularly significant. China’s import trade made up a significant amount of the world’s upstream products as of 2023—45%—nearly half of the worldwide market share. It indicates that the country’s enormous need for upstream items.

Lastly, the quantity of goods traded in each link of the photovoltaic business varies significantly. Upstream products have the smallest trade amount, making up less than 5% of the total trade amount of the photovoltaic industry chain. Downstream products have the largest trade amount, accounting for more than 70% of the total trade amount. Midstream products are the next most popular. Because upstream products have low trade prices despite having a large trade volume, which results in a relatively small proportion of trade volume. Downstream products have higher technical difficulties, production costs and prices, which results in a larger proportion of trade volume.

#### Analysis of trade flow in photovoltaic industry

**Fig. 4:**
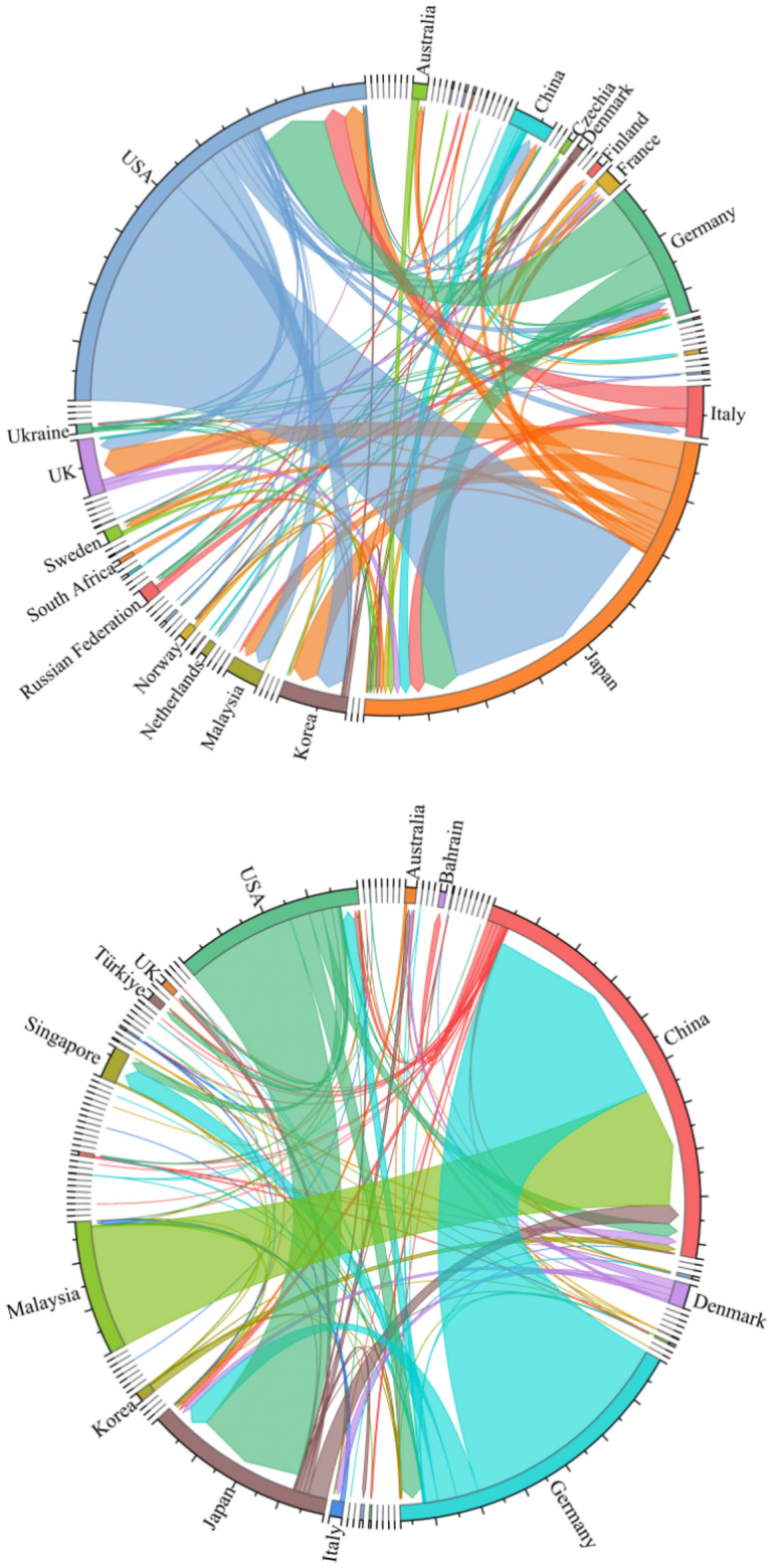
Upstream trade flow chart in 2000 and 2023.

A trade flow chart of the upstream photovoltaic business was created for the years 2000 and 2023 using global trade data from the UN Comtrade database. Figure 4 illustrates the polarization shift phenomena in the upstream product trade pattern. The two major trade patterns centered on the United States and Japan in 2000 are gradually shifting to two major trade patterns centered on China and Germany in 2023. As the largest producer in the world, China plays a significant role in the manufacture and export of polysilicon. However, some high-quality and high-purity polysilicon products still depend on imports because of technological deficiencies. Concurrently, the global market demand for polysilicon is increasing due to the fast expansion of industries like new energy and clean energy cars. Extraction cannot keep up with production’s consumption and use. China’s enormous need for polysilicon has caused it to steadily rise to the forefront of international trade. It now stands shoulder to shoulder with Germany as one of the core nations, shaping a new bipolar pattern of trade.

**Fig. 5:**
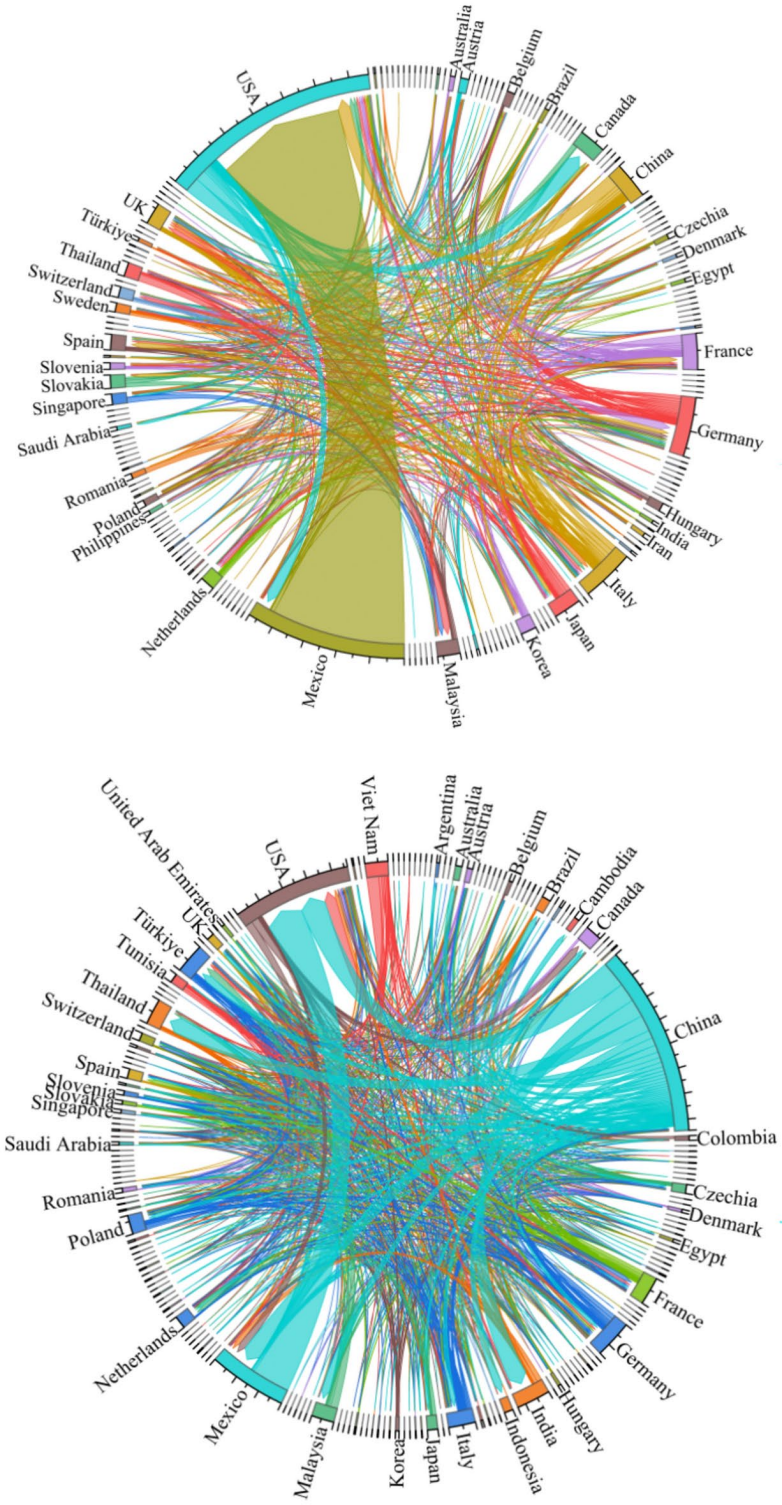
Midstream trade flow in 2000 and 2023.

In the pattern of trade in midstream products, the bipolar pattern led by the United States and Mexico is gradually disintegrating. A unipolar development pattern centered on China is gradually taking shape (see Fig. 5). China is the leading exporter of midstream commodities, with a concentration of exports to European and emerging nations. The demand for photovoltaic cells and their components has increased due to the new energy vehicle industry’s rapid development. Chinese companies constantly seek out other markets and boost their export volume while satisfying the demands of their home market. With their high-quality goods and extremely reasonable costs, Chinese photovoltaic firms have earned bigger market share, especially in places like ASEAN and Europe where there is a considerable demand for solar energy.

**Fig. 6:**
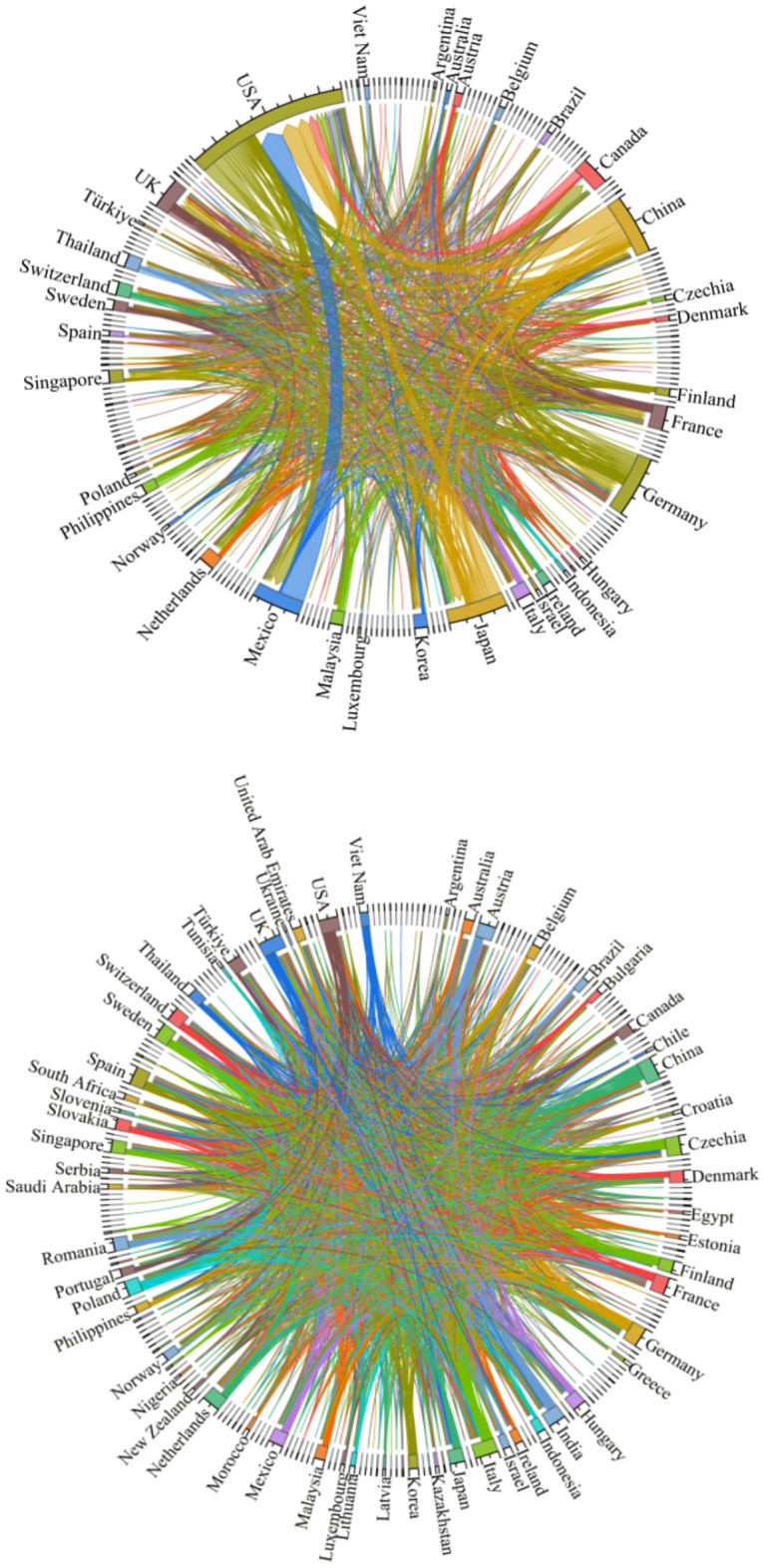
Downstream trade flow chart for 2000 and 2023.

The downstream product trade pattern exhibits a notable multipolarity trend (see Fig. 6). An increasing number of nations are engaging in the trade market. This is due to the fact that different nations and areas have various market demands and consumption patterns. Products need to be produced according to the specific conditions of each market. This promotes the decentralization of trade. In addition, technological progress and industrial upgrading have increased production efficiency and reduced costs. More countries and regions are able to produce competitive products and thus participate in international trade. This has also contributed to the decentralization of trade patterns.

### Kernel density estimation

The PageRank centrality of each link in 2000 and 2023 was determined using Python in order to precisely identify important nodes in the solar industry chain. The top 10% of node nations are displayed in Table 2 below. As shown in Table 2, the number of node countries involved in each link shows a small increasing trend. This indicates that the trade networks in the upper, middle and lower reaches of the PV industry chain are expanding. There have been notable alterations in the top 10% of nations with respect to PageRank centrality in every link, especially in upstream products. The PageRank centrality rankings of upstream products in US, UK, Italy and other European countries have continued to decline. It indicates a gradual decline in the position of US and other European countries in the industry chain. China’s ranking in the upstream,midstream and downstream links of the industrial chain has improved significantly. China’s influence is growing due to its ample resources and government policy support. Europe also holds a significant position in the upstream, midstream, and downstream of the industrial chain. With ongoing technological advancements, China’s photovoltaic industrial chain’s downstream development has demonstrated strong vitality and promising futures. China’s influence must still be reinforced and enhanced in the middle of the industrial chain. China should strengthen international cooperation and expand the development of midstream resources and technological innovation. Enhance its strategic position and core competitiveness in the global solar energy industry chain.

**Table 2:** PageRank centrality top 10% countries.

Year	Industry Chain	Node country
2000	Upstream (8)	Japan, USA, Germany, United Kingdom, Italy, China, Korea, Malaysia
	Midstream (17)	USA, Italy, Germany, Canada, France, Mexico, China, Malaysia, Japan, Thailand, United Kingdom, Netherlands, Singapore, Hungary, Korea, Slovakia, Romania
	Downstream (19)	USA, Germany, Japan, Canada, France, United Kingdom, Mexico, China, Netherlands, Italy, Switzerland, Malasia, Sweden, Singapore, Philippines, Korea, Thailand, Ireland, Austria
2023	Upstream (9)	Germany, China, Japan, USA, Singapore, Denmark, Italy, Korea, Bahrain
	Midstream (17)	China, USA, Viet Nam, Germany, Mexico, Canada, France, Türkiye, Poland, Italy, Tunisia, India, Cambodia, Thailand, Korea, Argentina, Netherlands
	Downstream (21)	Germany, USA, China, Netherlands, Mexico, France, Canada, Japan, United Kingdom, Viet Nam, Hungary, Italy, Korea, Thailand, Romania, Czechia, India, Poland, Spain, Slovakia, Austria

**Fig. 7:**
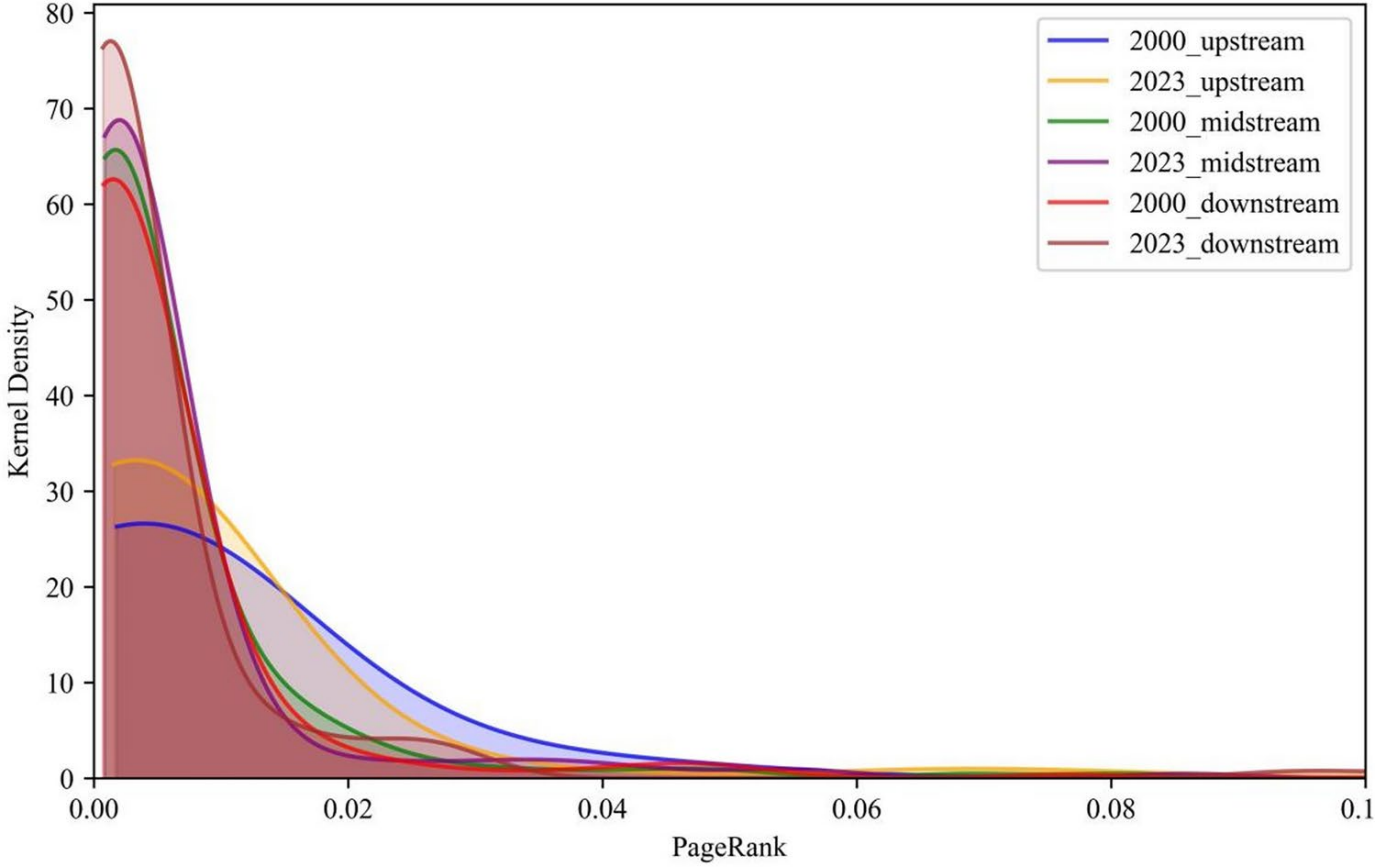
Kernel density analysis based on PageRank centrality.

The kernel density estimation curves of PageRank centrality for each stage in 2000 and 2023 were shown in order to examine the dynamic evolution trend of the PageRank centrality distribution (see Fig. 7). As a whole, the upper, middle and lower reaches are characterized by a single peaked distribution and a right trailing tail. These countries have high PageRank centrality and are at the center of the trade network. The core density estimation curves’ peak heights for upstream, midstream, and downstream products have all increased. This suggests that nodes with high PageRank centrality values have more control and influence over the network. Nuclear density curve of downstream products gradually shifts to the left. It shows that centrality value of PageRank in node countries decrease in downstream product. Kernel density curve of midstream products gradually shifts to the right. It indicates that centrality value of PageRank in node countries increase in midstream product.

### Vulnerability analysis of photovoltaic industry chain trade network

#### Evolution of vulnerability of photovoltaic industry chain trade network

**Fig. 8:**
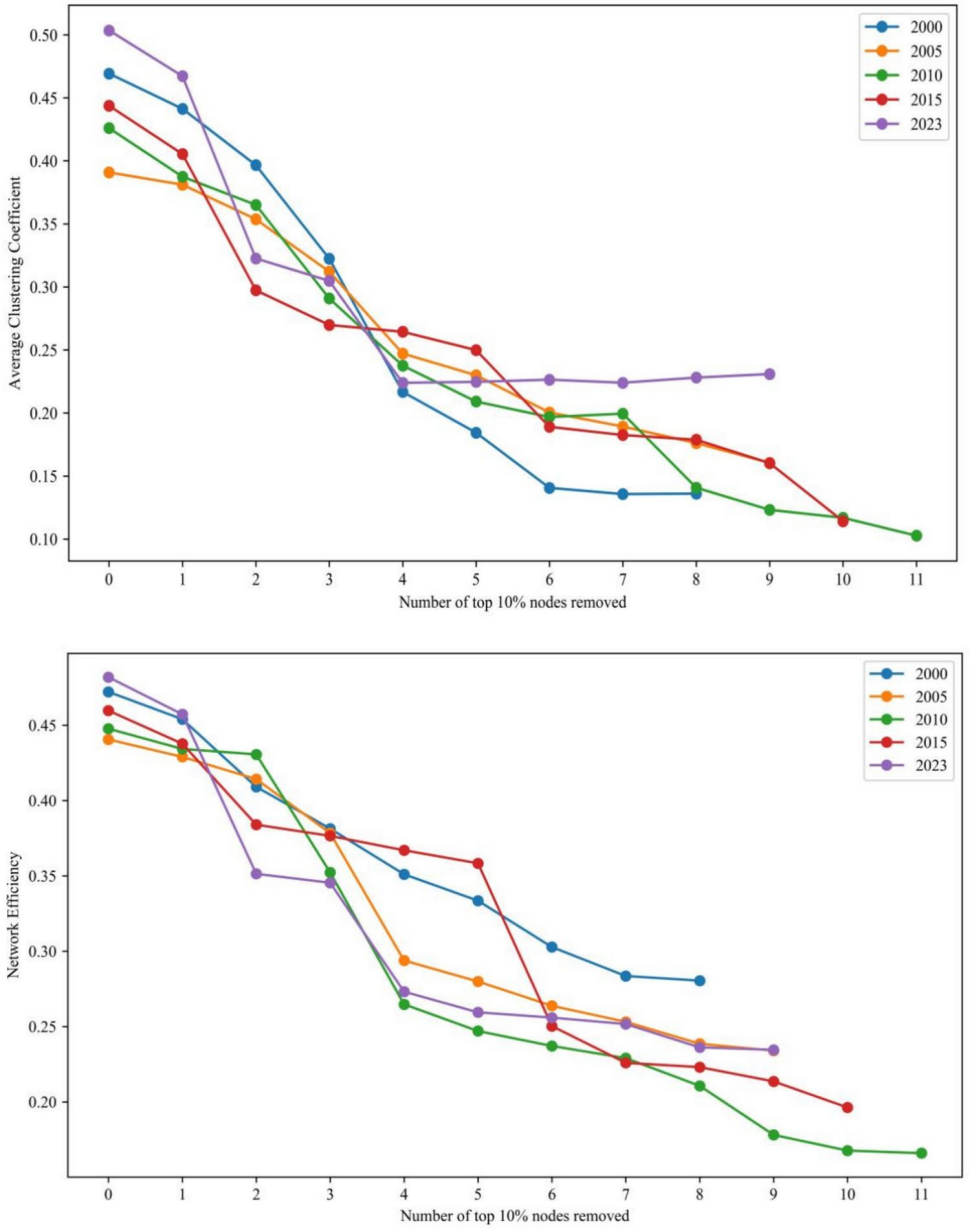
Comparison of changes in network characteristic values after intentionally attacking the top 10% nodes in the upstream of the photovoltaic industry chain.

**Fig. 9:**
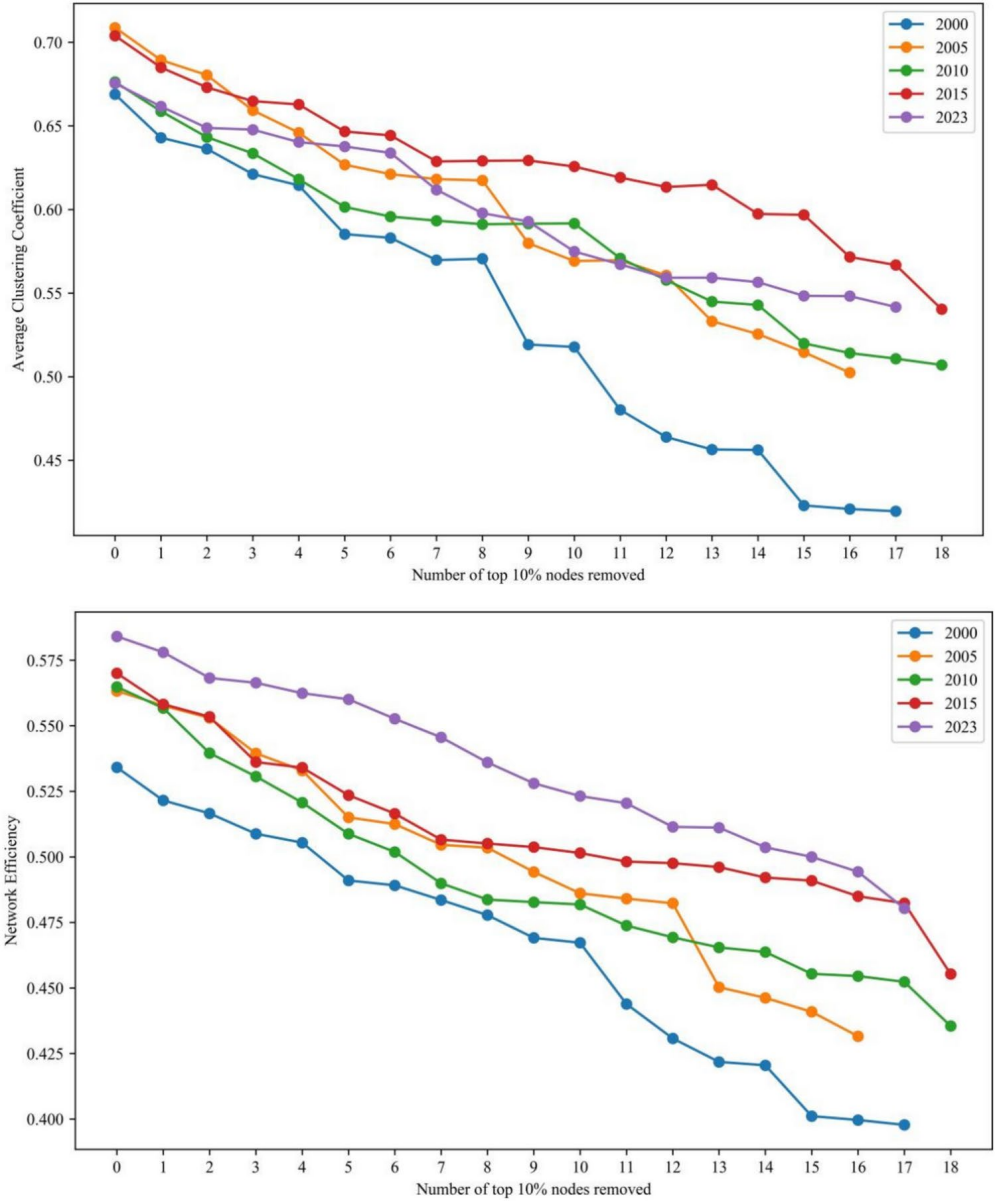
Comparison of changes in network characteristic values after intentionally attacking the top 10% nodes in the midstream of the photovoltaic industry chain.

**Fig. 10:**
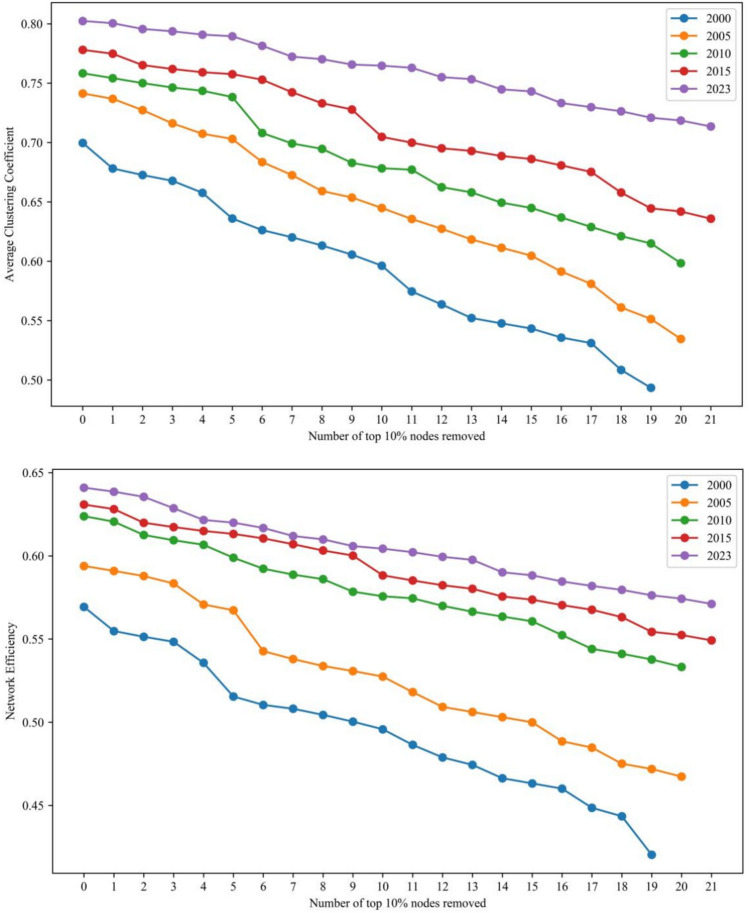
Comparison of changes in network characteristic values after intentionally attacking the top 10% nodes in the downstream of the photovoltaic industry chain.

A line graph was created to illustrate the changes in network eigenvalues following the gradual removal of the top 10% nodes in PageRank centrality in 2000, 2005, 2010, 2015 and 2023. The graphs can better explore the evolution of network susceptibility under trade interruption. Based on a comparison analysis, the subsequent findings were obtained:

1. The photovoltaic manufacturing chain’s upstream network is rather steady (Fig. 8). The upstream network is now more tightly clustered, which suggests that the network’s tightness has risen. More specifically, when the percentage of failing nodes rises, network efficiency and clustering both decline. In 2000 and 2023, the network characteristic values saw yearly average growth rates of − 47.39%, − 26.31% and − 45.88%, − 38.56%, respectively. Network aggregation and efficiency have a negative impact on network vulnerability in the upstream of the PV chain.
2. The midstream network of the PV industry chain is more stable (Fig. 9). Network aggregation and network efficiency decrease with increasing percentage of failed nodes. Network characteristic values saw yearly average growth rates of − 19.71%, − 12.31% and − 11.59%, − 8.97% in 2000 and 2023. In the solar industrial chain, midstream networks’ vulnerability is negatively impacted by network clustering and efficiency.
3. The downstream network resistance of the PV industry chain has improved, and the fluctuation of node impacts on the network structure has weakened (Fig. 10). Network aggregation and efficiency decrease with the increase of the proportion of failed nodes. The annual average growth rates of network characteristic values in 2000 and 2023 are − 15.57%, − 13.41% and − 5.29%, − 6.13%, respectively. Network aggregation and efficiency also have a negative impact on network vulnerability downstream of the PV industry chain.

In conclusion, compared to 2000, the trade network of each link of the PV industry chain is more stable in 2023. There is a slight increase in network destruction resistance. The impact of node failure on the network structure is reduced. All these indicate that, after 24 years of development and evolution, the trade network of each link of the PV industry chain is gradually moving towards a new stage that is more robust and reliable.

#### The impact of nodes on the vulnerability of the photovoltaic industry chain trade network

**Fig. 11:**
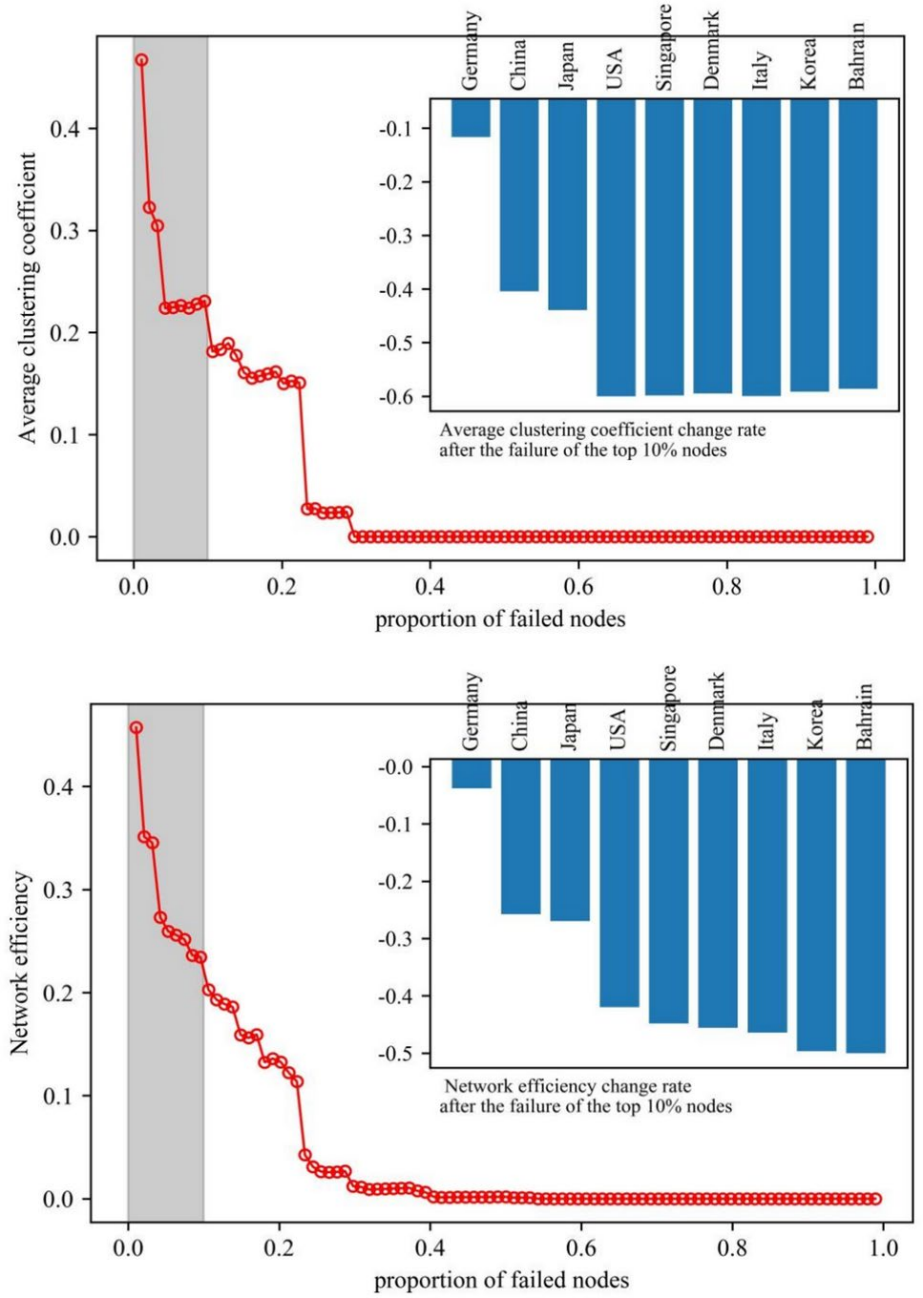
Vulnerability analysis of upstream networks in the photovoltaic industry chain.

**Fig. 12:**
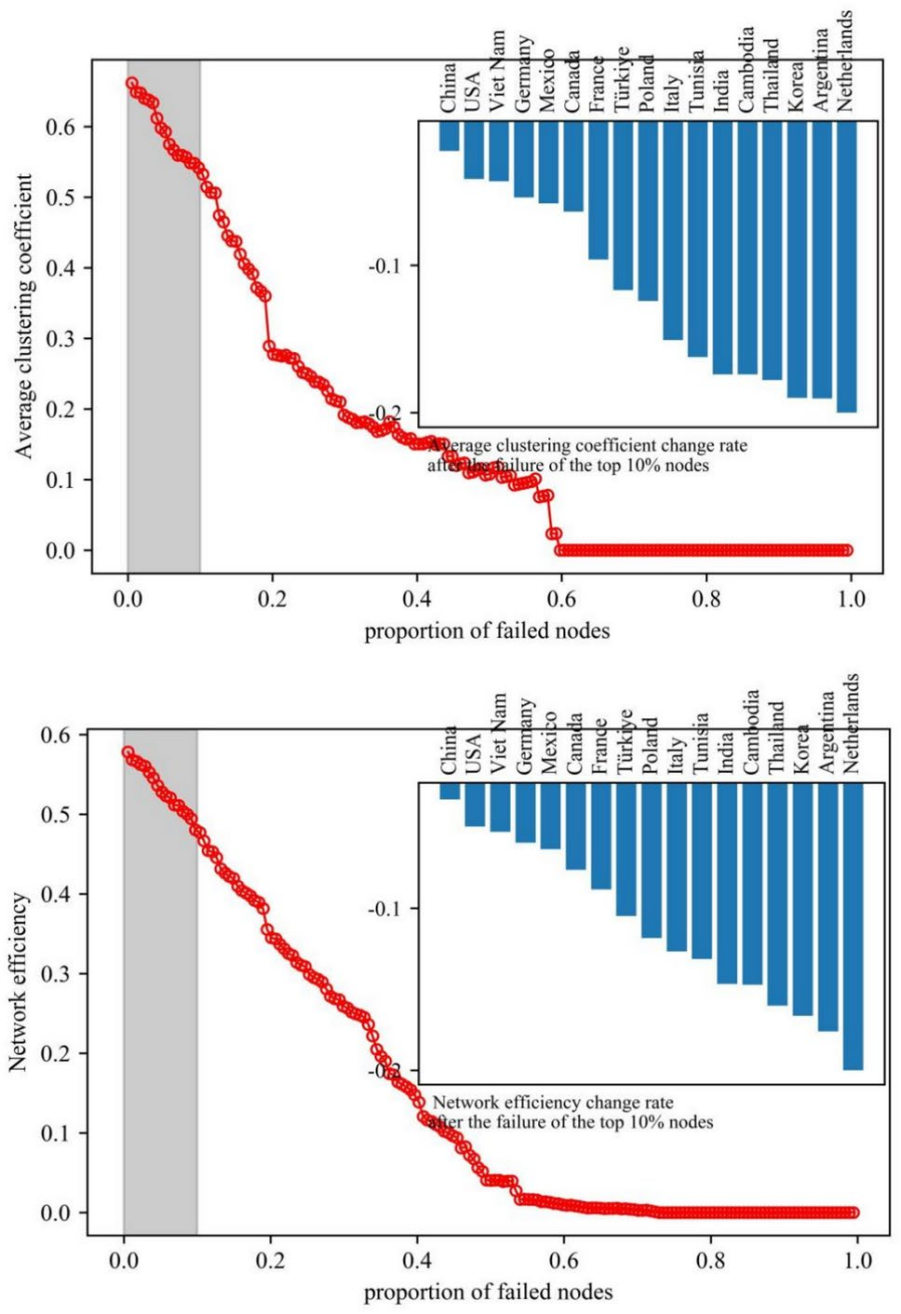
Vulnerability analysis of midstream networks in the photovoltaic industry chain.

**Fig. 13:**
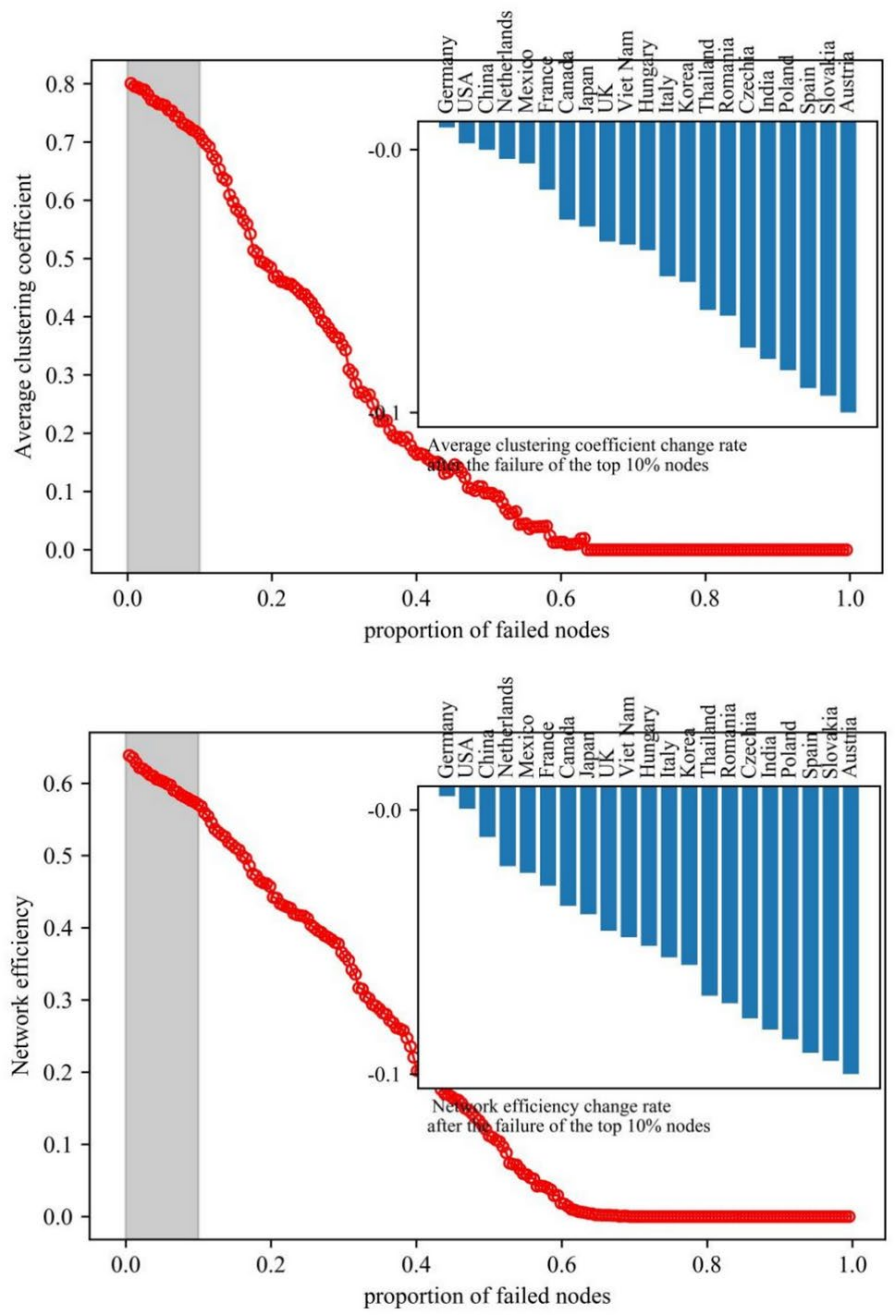
Vulnerability analysis of downdstream networks in the photovoltaic industry chain.

Vulnerability analysis charts were created for networks of the photovoltaic industry chain in order to investigate the impact of nodes on the vulnerability of the trade network. The charts can reflect changes in network vulnerability of each link in the photovoltaic industry chain under deliberate attacks and measure the rate of change in network characteristic values after key node failures (see Figs. 11, 12, and 13). Overall, the clustering and efficiency of networks show a strong power-law decay distribution pattern as the fraction of failing nodes rises. The distribution of upstream resources and extraction technologies are concentrated in a few countries. Therefore, the high concentration of resources and the monopoly of technology make the upstream network show solidification effect. When some key nodes fail, the characteristic value of the network changes significantly. The instability leads to a significant increase in the vulnerability of the network.

Network agglomeration reflects the closeness of nodes. The greater the agglomeration, the greater the possibility for countries to jointly cope with and defend against risks by means of cooperation. ① The agglomeration of the upstream network shows a stepwise decline, with an average decline of 54.15%. The failure of nodes in Vietnam, the United States, and Germany has less impact on the network agglomeration. Until the failure of the nodes in China, the agglomeration of the network decreases significantly. When the proportion of failed nodes is roughly 27%, the network faces collapse. ② The agglomeration of the midstream network shows a breakpoint decline, and the overall decline is 19.41%. The failure of nodes in China, Lebanon, the United States, the Philippines, Germany, and India has a more significant impact on the network agglomeration. When the proportion of failed nodes is about 58%, the network faces collapse. ③ The agglomeration of the downstream network shows a continuous and uniform decreasing trend, and the overall decrease is 10.74%. When the proportion of failed nodes is nearly 63%, the network faces collapse. In addition, the failure of key nodes affects the upstream network agglomeration to the greatest extent.

Network efficiency reflects the efficiency of commodity information transmission. The higher the value of network efficiency, the more rapidly information or resources may be disseminated in the network. ① The efficiency of the upstream network decreases in a stepwise manner, with an average decrease of 51.34%. The failure of nodes in Vietnam, the United States, and Germany had little effect on the network’s efficiency. Until the failure of Chinese nodes, the efficiency of the network decreases significantly. When the proportion of failed nodes is nearly 30%, the network faces collapse. ② The efficiency of the midstream network shows a continuous and uniform decreasing trend, with a decrease of 17.86%. When the proportion of failed nodes is roughly 53%, the network faces collapse. ③ The efficiency of the downstream network shows a continuous and uniform decreasing trend, and the decreasing rate is about 10.94%. When the proportion of failed nodes is 62%, the network faces collapse. The failure of key nodes has the greatest degree of influence on the efficiency of the upstream network.

## Conclusion and suggestions

### Conclusion

The intensification of economic globalization has led to closer trade relations between countries. It has resulted in a vast and complex network system. The riskiness and vulnerability of the network also come with it. This paper constructs the global photovoltaic industry chain trade network from 2000 to 2023 based on the complex network analysis method. The change of network vulnerability in multiple years was comparatively analyzed. The influence of key nodes on network vulnerability was examined. The conclusions are drawn as follows:

1. The trade pattern of the photovoltaic industry chain has undergone profound changes. The phenomenon of upstream polarization shift is obvious, forming two major trade patterns with China and Germany as the core. The midstream trade pattern shows a centralized distribution trend, with China occupying an absolute core position in the trade network. Downstream trade is more dispersed, with a significant trend towards multi-polarization.
2. The network vulnerability of the PV industry chain shows a weakening trend. The development of upstream and midstream networks is more stable. The eigenvalue of upstream network agglomeration has increased, indicating that the tightness of upstream network has increased. The destruction resistance of the downstream network has increased. The fluctuation of node impact on network structure is weakened. Both network agglomeration and network efficiency have a negative impact on the vulnerability of upstream, midstream and downstream networks in the PV industry chain.
3. The vulnerability ranking of PV industry chain is: downstream < midstream < upstream. When the proportion of failed nodes is about 30% or so, the upstream network faces collapse. The proportion of nodes facing collapse in midstream and downstream networks is 58% and 60% respectively. When the top 10% nodes of PageRank centrality fail, the agglomeration of upstream, midstream and downstream network decreases by 54.15%, 19.41%, 10.74% and the efficiency of the network decreases by 50%, 14% and 7.5% respectively.

### Policy recommendations

Drawing from the aforementioned research findings, this article puts forth the following policy recommendations to guarantee the security of the global solar industry chain:

1. Based on the trend of the evolution of the trade network pattern of the global photovoltaic industry chain, the diversification of trade partners should be promoted in the future. Focus on emerging markets with growth potential. Accelerate the construction of Free Trade Area(FTA). Negotiate high-standard FTA with more countries. Support industry organizations and trade promotion agencies to build public service platforms.
2. In view of the changes in the vulnerability of the trade network of each link in the photovoltaic industry chain under trade disruption, a risk early warning and response mechanism should be established. Accurately identify the weak links of the industrial chain. Monitor potential risk points in real time, make a good risk assessment, and prepare a response plan in advance according to the actual situation.
3. According to the vulnerability ranking of the PV industry chain, emphasis should be placed on safeguarding the global allocation capacity of upstream silicon materials. Countries should continue to increase capacity investment and develop new silicon resources. Adjust the production capacity according to the market demand of each country and optimize the industrial layout. At the same time, they should increase investment in technology research and development, and enhance production capacity and production efficiency through technological innovation and transformation.

## Data Availability

All raw data in this article are sourced from UN Comtrade (https://comtradeplus.un.org/). The data that support the findings of this study are available from the corresponding author, [Xiaobei Ren], upon reasonable request.
